# Autophagy Mediates MMP-2 Expression in Glaucomatous Trabecular Meshwork Cells

**DOI:** 10.1155/2022/6026464

**Published:** 2022-09-10

**Authors:** Yan-Ling Xiao, Xiao-Rui Wang, Kejing Ye, Wan-Zhu Chen, Bing-Ru Zheng, Yi-Hong Huang, Yu-Yu Wu

**Affiliations:** Department of Ophthalmology, The Second Affiliated Hospital of Fujian Medical University, Quanzhou 362000, Fujian Province, China

## Abstract

**Purpose:**

To investigate the effect of 3-methyladenine (3-MA) and starvation on the expression of matrix metalloproteinase (MMP-2) in patients with primary open-angle glaucoma.

**Methods:**

Primary TM cells were cultured and divided into three groups. The control group was treated with a normal medium, the 3-MA group was stimulated with 3-MA, and the starvation group received nutrient depletion by replacing the normal media with Earle's balanced salt solution. Cellular mRNA and protein were measured at different 3-MA concentrations and starvation time periods. The level of autophagy was accessed by monodansylcadaverine fluorescent staining and expression of specific autophagy-related genes, light chain 3 (LC3), and Beclin1. The effects of 3-MA and starvation on cell proliferation were determined with a 3-(4,5-dimethylthiazol-2-yl)-2,5-diphenyl tetrazolium bromide assay kit. The mRNA and protein expression of LC3-II, Beclin1, and MMP-2 were measured by reverse transcription-polymerase chain reaction and western blot, respectively.

**Results:**

Compared to the control group, starvation significantly upregulated LC3-II and Beclin1 in TM cells after 3 h of stimulation, which peaked at 6 h and 9 h, respectively. Increased MDC-labeled cells were also observed. Starvation downregulated the expression of MMP-2. On the contrary, 3-MA suppressed the activation of autophagy, as shown by the marked downregulation of LC3-II and Beclin1. The expressions of MMP-2 were higher in the 3-MA group compared to the control group, reaching a peak at a concentration of 5 mM.

**Conclusion:**

Autophagy may be involved in the pathogenesis of POAG via regulating the expression of MMP-2 and, subsequently, the deposition of the extracellular matrix.

## 1. Introduction

Primary open-angle glaucoma (POAG) is a degenerative disease characterized by progressive retinal ganglion cell (RGC) loss and typical visual field defects [1]. Although the precise molecular mechanism of POAG is far from being understood, elevated intraocular pressure (IOP) remains the major risk factor as well as the sole target for clinical intervention [2]. Elevated IOP occurs when there is excessive resistance to aqueous humor (AH) drainage through the trabecular meshwork (TM), the primary outflow tract [3]. TM cells are responsible for establishing and monitoring AH outflow resistance and maintaining IOP homeostasis [3, 4]. Numerous previous studies suggested that accelerated production of reactive oxygen species (ROS) leads to oxidative damage to the TM cells, which might contribute to the development of POAG, and one of the main catabolic pathways for degrading these cellular materials is autophagy-lysosomal (AL) pathways [5, 6]. Autophagy is emerging as an essential cellular survival mechanism against a variety of stressors including oxidative stress, starvation, and mechanical forces [5, 7]. Impaired lysosomal degradation in oxidatively stressed cultures has also been implicated in the pathogenesis of POAG [8].

Most of the outflow resistance at the anterior chamber angle is thought to come from the extracellular matrix (ECM) of the juxtacanalicular region, the deepest portion of the TM [9, 10]. Matrix metalloproteinases (MMPs) belong to the metzincin clan of the metalloproteinase superfamily and are named after their ability to cleave and remodel the ECM [11]. In the TM, certain MMPs are highly expressed during resting conditions, and these may function to maintain the open outflow pathway [12]. A study by Porter et al. [13] found that autophagy was activated in the TM cells in an mTOR-independent manner in response to the static biaxial stretch and in high-pressure perfused eyes. It is also suggested that in cases with IOP elevation, TM cells could sense increased mechanical stretching forces and respond by regulating the secretion of MMPs and the tissue inhibitor of metalloproteinase (TIMP)-2 to increase the trabecular ECM turnover rate, reduce the aqueous outflow resistance, and restore normal IOP [14, 15].

We hypothesized that in the TM cells, the level of autophagy may be involved in the pathogenesis of POAG via regulating the expression of MMPs and deposition of ECM. Thus, in this study, we investigated the effects of 3-MA and starvation on the expression of MMP-2 in cultured TM cells from POAG patients.

## 2. Methods

### 2.1. Primary Trabecular Meshwork Cell Culture

Primary TM cells (TMCs) were isolated and cultured as described previously [16]. In brief, the TM tissues were dissected from several POAG patients aged 30 to 52 years who underwent trabecular surgery without using mitomycin C at the Second Affiliated Hospital of Fujian Medical University, Fujian, China, from October 2015 to April 2017. This study has received approval from the Ethics Committee of Fujian Medical University, Quanzhou, China, and written informed consent was obtained from each participant. All the experiments were conducted in accordance with the tenets of the Declaration of Helsinki. The collected trabecular tissues were cultured in DMEM/F12 (1 : 1 ratio of Dulbecco's Modified Eagle's Medium and Ham's F12 medium; Hyclone, Logan, UT) containing 20% fetal bovine serum (FBS; Hyclone) and 1% penicillin-streptomycin-gentamicin (Hyclone) in a humidified environment of 5% CO_2_ at 37°C. Cell growth was monitored regularly with an inverted microscope (Olympus, Japan). Prior to reaching 80% confluence, a 0.25% concentration of trypsin was used for cell subculture. Primary TM cells were used for most of passage five.

### 2.2. Immunocytochemical Staining

Cell identification was performed as described previously [17]. In brief, the third-generation TM cells were plated in six-well plates, and the cells were processed for immunocytochemical staining with mouse monoclonal antihuman fibronectin (FN), laminin (LM), neuron-specific enolase (NSE), factor VIII-associated antigen, and vimentin antibodies (New Step Company, Fuzhou, China). They were visualized with an inverted fluorescent microscope (Olympus, Tokyo, Japan).

### 2.3. Study Intervention

The cultured cells were divided into three groups: the 3-MA group, the starvation group, and the control group. Cells in the 3-MA group were stimulated with the autophagy inhibitor 3-methyladenine (3-MA; Sigma-Aldrich, St. Louis, MO) for 24 h. According to the recommendation, 3-MA was dissolved in the PBS with gentle heating, reconstituted at a concentration of 100 mM, and diluted to the working concentration (1.25, 2.5, 5, and 10 mM) with a fresh medium. A starvation model was established in the starvation group by replacing the normal media with Earle's balanced salt solution (EBSS; Sigma) for 3, 6, 9, and 12 h to stimulate nutrient depletion. Cells in the control group were treated with an equivalent normal medium. Notably, the working concentrations of 3-MA and time points of starvation were chosen based on previous reports [18, 19].

### 2.4. MTT Cell Viability Assays

The effects of 3-MA and starvation on cell proliferation were determined with a 3-(4,5-dimethylthiazol-2-yl)-2,5-diphenyl tetrazolium bromide (MTT) assay kit (KeyGEN Biotechnology, Nanjing, China). The cells were incubated in 96-well culture plates (Corning, NY) at a concentration of 1 × 10^4^ cells/well and divided into the following three groups: the 3-MA group (0, 2.5, 5, 10, and 20 mM), the starvation group (6, 12, 18, 24, and 36 h), and the remaining cells without any intervention, which was used as the control group.

### 2.5. Monodansylcadaverine (MDC) Fluorescent Staining

The fluorescent staining was performed according to the method described previously by Contento et al. [20]. In brief, cells were incubated on a round slide in a 6-well plate until reaching 50–60% confluence. As described above, they were divided into three groups to receive different interventions. When arriving at corresponding time points, the slides were washed, and 0.05 mM MDC was added to each slide. The slides were then placed in the incubator for 1 h, protected from light. After being fixed with 4% paraformaldehyde for 15 min, the slides were washed and then mounted by ultraviolet light. Finally, they were observed by inverted immunofluorescence microscopy.

### 2.6. Reverse Transcription-Polymerase Chain Reaction (RT-PCR)

Procedures for RNA extraction and RT-PCR have been described previously [17]. RNA was extracted from all groups of TM cells using the Trizol reagent (Life Technologies, Carlsbad, CA). First-strand cDNA was synthesized from RNA by reverse transcription using an oligo (dT) primer and reverse transcriptase (Takara, Tokyo, Japan). Real-time PCR analysis was performed using the Prime-Script RT reagent kit with SYBR Green (Takara) on an Applied Biosystems 7500 Real-Time PCR System according to the manufacturer's instructions. The cycling parameters were set at 95°C for 30 s (denature), 95°C for 5 s (annealing), and 60°C for 34 s (extension), and 40 cycles were performed. *β*-Actin served as an internal standard of mRNA expression. Relative quantification was calculated with the formula 2^−ΔΔCt^, where ΔCt = Ct_gene_−Ct_Act_ and ΔΔCt = ΔCt_Exp_−ΔCt_Con_. The sequences of the primers used for the amplifications are shown in Table 1.

### 2.7. Western Blot Analysis

Cellular protein was harvested after treatment, washed twice with cold phosphate-buffered saline (PBS; Hyclone, Logan, UT) solution, and lysed in ice-cold radioimmunoprecipitation (RIPA; Beyotime Institute of Biotechnology, Shanghai, China) buffer containing 100 mM phenylmethylsulfonyl fluoride (PMSF; Beyotime) which was added to extract the total proteins. All steps were carried out on ice if possible. Lysates were centrifuged at 12,000 rpm at 4°C for 15 min, and then the supernatant was collected. Protein concentration was quantified with a bicinchoninic acid (BCA; Beyotime) assay. The proteins were separated using 10% sodium dodecyl sulfate-polyacrylamide gel electrophoresis (SDS-PAGE; Beyotime). After electrophoresis, the proteins were transferred onto polyvinylidene fluoride (PVDF; Millipore, MA, USA) membranes. The membranes were subsequently blocked with 5% skimmed milk dissolved in Tris-buffered saline Tween (TBST; Beyotime) for 2 h and incubated with the primary antibody at 4°C overnight. The primary antibodies included anti-LC3B (Cell Signaling Technology, Beverly, MA), anti-Beclin1 (CST), anti-MMP-2 (Abcam, Cambridge, UK), and anti-*β*-actin (Boster Biological Technology, Wuhan, China). Then, the membranes were washed with TBST three times, followed by incubation with the horseradish peroxidase-linked secondary antibody (Boster) at a concentration of 1: 5,000 for 1 h at room temperature. The blots were visualized with an enhanced chemiluminescence kit (Amersham, ECL Plus, Freiburg, Germany), and the images were collected and analyzed by Quantity One (Bio-Rad, CA, USA).

### 2.8. Statistical Analysis

All experimental procedures were repeated three times, and the data are presented as the mean ± standard deviation (SD). The statistical analysis was performed using SPSS 21.0 software (SPSS Inc., Chicago, IL), and a one-way analysis of variance (ANOVA) was used to test the differences among different groups. A *p* value of less than 0.05 was considered statistically significant.

## 3. Results

As shown by the inverted microscope, the cultured cells demonstrated varying shapes, including stelliform, ellipse, triangle, and irregular. Immunocytochemical staining suggested that fibronectin (FN; SFigure 1A), laminin (LM; SFigure 1B), neuron-specific enolase (NSE; SFigure 1C), and vimentin (SFigure 1D) were all positive, while factor VIII (SFigure 1E) and negative control staining (SFigure 1F) were not positive. Both the morphological and immunocytochemical examinations confirmed that the cultured cells were TM cells.

The MTT assay revealed that both 3-MA and starvation could inhibit the proliferation of TM cells. As shown in SFigure 2A, the effect of 3-MA is dose-dependent, and the inhibition rate increased significantly when the cells were incubated with a 10 mM or higher concentration of 3-MA for 24 h. Also, stimulating for 48 h demonstrated a higher inhibition rate than for 24 h at all 3-MA concentrations. The effect of starvation on the cell inhibition rate is time-dependent and significantly increased when exceeding 12 h (SFigure 2B).

**Figure 1:**
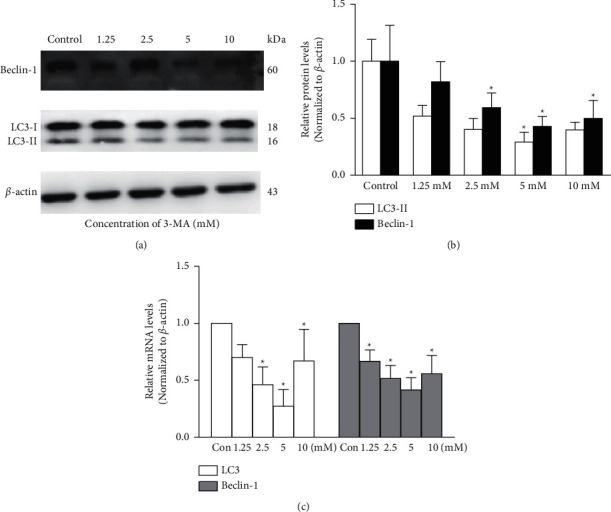
3-MA inhibits the expression of Beclin-1and LC3 in cultured trabecular meshwork cells. (a) Beclin-1 and LC3 expressions were detected with western blotting in trabecular meshwork cells following different concentrations of 3-MA (mM). (b) The histogram shows densitometric analysis of average Beclin-1 and LC3 levels. *β*-Actin was used as an internal control. Cells without any intervention are used as a negative control. (c) Beclin-1 and LC3 expressions were detected with the reverse transcription-polymerase chain reaction (RT-PCR) in trabecular meshwork cells following different concentrations of 3-MA (mM). ^*∗*^*P* < 0.05.

**Figure 2:**
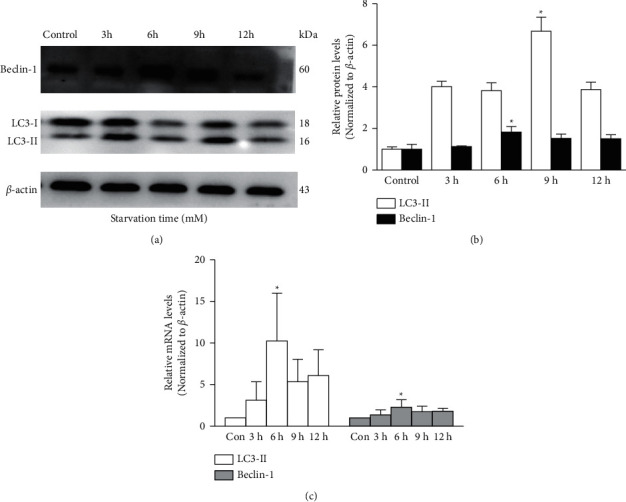
Starvation promotes the expression of Beclin-1 and LC3 in cultured trabecular meshwork cells. (a) Beclin-1 and LC3 expressions were detected with western blotting in trabecular meshwork cells following different starvation times (h). (b) The histogram shows densitometric analysis of average Beclin-1 and LC3 levels. *β*-Actin was used as an internal control. Cells without any intervention are used as a negative control. (c) Beclin-1 and LC3 expressions were detected with the reverse transcription-polymerase chain reaction (RT-PCR) in trabecular meshwork cells following different starvation times (h). ^*∗*^*P* < 0.05.

As shown in SFigure 3, as compared to the control group, the number of MDC-labeled cells was lower in the 3-MA group but higher after 6 h of nutrient depletion in the starvation group. A dose-response decrease in the protein levels of LC3-II/LC3-I ratio and Beclin-1 was found in the 3-MA group as compared to the control group. The LC3-II and Beclin-1 protein expressions were downregulated by 29 ± 8% (*p* = 0.046) and 43 ± 9% (*p* = 0.009) at 5 mM of 3-MA, respectively (Figure 1(b)). The RT-PCR results were consistent with the western blot analysis. As expected, the LC3 mRNA level decreased by 73 ± 14%, and the Beclin-1 mRNA level decreased by 41 ± 10% in the TM cells at 5 mM of 3-MA when compared to the control group (Figure 1(c)). In contrast, the LC3-II and Beclin1 protein levels increased over time and peaked at 9 h (6.67 ± 0.67-fold, *p* = 0.042) and 6 h (1.83 ± 0.26-fold, *p* = 0.018) of starvation, respectively, in the starvation group as compared to the control group (Figure 2(b)). The PCR results provided further evidence that LC3 and Beclin-1 were upregulated after starvation by EBSS at 6 h (10.25 ± 5.74-fold and 2.29 ± 0.89-fold, respectively, *p* < 0.05, Figure 2(c)).

**Figure 3:**
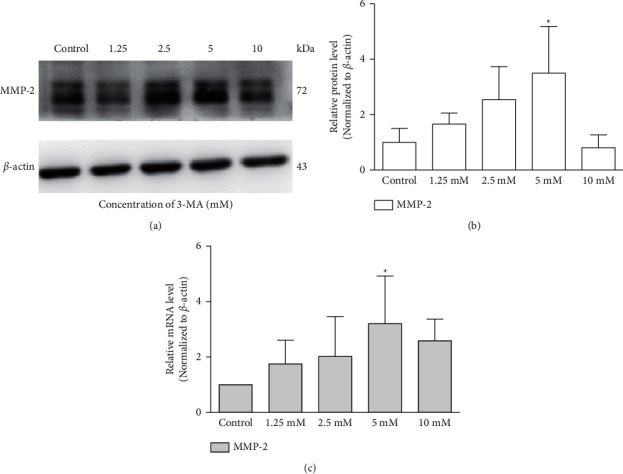
3-MA promotes the expression of MMP-2 in cultured trabecular meshwork cells. (a) MMP-2 expression was detected with western blotting in trabecular meshwork cells following different concentrations of 3-MA (mM). (b) The histogram shows densitometric analysis ofMMP-2 average levels. *β*-Actin was used as an internal control. Cells without any intervention are used as a negative control. (c) MMP-2 expression was detected with the reverse transcription-polymerase chain reaction (RT-PCR) in trabecular meshwork cells following different concentrations of 3-MA (mM). ^*∗*^*P* < 0.05.

**Figure 4:**
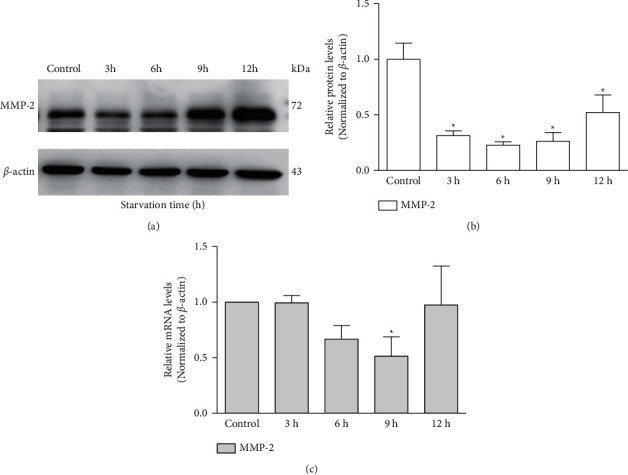
Starvation inhibits the expression of MMP-2 in cultured trabecular meshwork cells. (a) MMP-2 expression was detected with western blotting in trabecular meshwork cells following different starvation times (h). (b) The histogram shows densitometric analysis of MMP-2 average levels. *β*-Actin was used as an internal control. Cells without any intervention are used as a negative control. (c) MMP-2 expression was detected with the reverse transcription-polymerase chain reaction (RT-PCR) in trabecular meshwork cells following different starvation times (h). ^*∗*^*P* < 0.05.

Western blotting revealed that supplementation with 3-MA resulted in a dose-response elevation of MMP-2 protein levels in the TM cells as compared to the control group, reaching a peak at 5 mM of 3-MA (1.83 ± 0.26-fold, *p*=0.034, Figure 3(b)), which returned to almost baseline levels at 10 mM of 3-MA. The results of RT-PCR showed that the mRNA level of MMP-2 significantly increased by 3.20 ± 1.71-fold with the treatment of 3-MA (5 mM) compared with the control group (*p*=0.037, Figure 3(c)). On the contrary, MMP-2 protein expression decreased by 77 ± 3% under starvation at 6 h (*p*=0.001, Figure 4(b)), and the MMP-2 mRNA level decreased by 51 ± 17% at 9 h compared to the control group (*p*=0.009, Figure 4(c)).

## 4. Discussion

Despite the fact that glaucoma is one of the leading causes of irreversible blindness worldwide, little is known about its underlying pathogenesis, and there are currently no biomarkers that could help clinicians with accurate early diagnosis [21, 22]. In this study, we found a dose-response association between 3-MA and starvation treatment with MMP-2 expression in cultured TM tissues from POAG patients, suggesting that autophagy may be related to POAG pathogenesis via regulating the expression of MMP-2.

The TM can interfere with the IOP by regulating the AH outflow [3]. At present, the mechanism of apoptosis of TM cells in POAG is not clear. Studies show that multiple mechanisms are involved in the pathological process of apoptosis of TM cells, including over-activation of endoplasmic reticulum stress, oxidative stress, and lack of trophic factors [4, 10, 22]. A growing number of evidence showed a role for autophagic dysregulation in the glaucomatous outflow pathway [4, 10, 23]. MMPs have been proved to play key roles in the degradation of gelatin, type IV collagen, and type V collagen, the major constituents of the ECM [24]. Also, cumulative evidence has suggested that autophagy plays an important role in the pathogenesis of cardiovascular diseases and tumors via the regulation of MMP-2 and MMP-9 [25, 26]. The MMPs are known as critical modulators of TM architecture and are promising therapeutic targets for restoring balanced outflow resistance. It is thus speculated that activation of autophagy might be involved in the pathogenesis of POAG through the regulation of MMP-2.

Microtubule-associated protein 1 light chain 3 (LC3) is synthesized as a precursor form cleaved by the protease ATG4B, and a key event required for autophagosome formation is the lipidation of the autophagosome marker LC3-I to LC3-II [27]. Therefore, the activation of autophagy is characterized by a dramatic elevation in the level of LC3-II, which could be prevented by pharmacological blockage of autophagosome formation with 3-methyladenine (3-MA) [15]. Beclin1, the first downstream autophagy execution gene, is a critical component in class III PI3 kinase (PI3KC3) that induces autophagosome formation [28, 29]. In this study, autophagy inhibition was applied using our well-established experimental model consisting of culturing confluent monolayers of the TM cells to 3-MA. A model of starvation induced to activate autophagy has been successfully established in various types of cancer cells such as prostatic cancer, lung cancer, and liver cancer but has not been reported in TM cells [30, 31]. We showed that both Beclin1 and LC3-II expression in the primary cultured TM cells decreased with 3-MA treatment in an almost dose-dependent manner, while they increased in a nearly time-dependent manner in the starvation-induced model. More interestingly, the LC3-II mRNA increase reached its peak after the 6 h starvation, while the LC3-II protein increase was more obvious at 9 h. A possible reason may be that protein synthesis takes more time due to its complicated as well as time-consuming processes, including DNA transcription, mRNA translation, and posttranslational modification of protein trafficking and secretion.

In this study, we showed that 3-MA upregulated MMP-2 expression in cultured glaucomatous TM cells. On the contrary, starvation decreased the expression of MMP-2. It remains unclear how the expression of MMP-2 is regulated by autophagy in POAG eyes. Li et al. [32] have reported that Atg5 knocking down could inhibit autophagy and increase the expression of MMP-2, mTOR, and p70S6K in endothelial progenitor cells (EPCS), which could result in significantly increased cell migration in wound healing and the trans-well assay under normoxic conditions, while rapamycin can enhance autophagy and attenuate the expression of MMP-9, mTOR, and p70S6K. However, little is known about the signal transduction pathways that mediate their effects. Evidence from previous studies suggests that the mTOR-p70S6K pathway plays an important role in cell migration [33, 34]. The importance of the mTOR-p70S6K pathway is additionally supported by the observation that the mTOR-p70S6K pathway could promote MMPs and uPA expression, which could facilitate cell migration by degrading ECM [35]. Furthermore, a recent work conducted by Luo et al. [35] also provided experimental evidence that gartanin significantly induced autophagy in T98G cells, while the secretion and activity of MMP-2 were significantly suppressed. In addition, the antiproliferation effect of gartanin in T98G glioma cells is most likely modulated by autophagy, which is regulated by the PI3K/AKT/mTOR signaling pathway. Therefore, autophagy may exert an antimigration effect on trabecular meshwork cells by inhibiting the expression of MMP-2, which is involved in the MAPK signaling pathway.

In addition to the generally used therapeutic and surgical treatments that could regulate the AH outflow, controlled overexpression of MMPs via gene therapy to restore the endogenous balance might be an efficient, long-lasting, and less invasive alternative. As we described above, available experimental evidence supports a key role of autophagy in maintaining outflow pathway tissue homeostasis. Nevertheless, further studies are also needed to compare the effect of autophagy levels on MMP-2 expression between normal controls and POAG patients.

Taken together, our study demonstrated that the expression of MMP-2 in the cultured glaucomatous TM cells was regulated by the autophagy level. We infer that the dysregulation of autophagy may cause the deposition of ECM and thus increase the resistance of AH outflow through MMP-2 and eventually result in IOP increase. This study provides a new line of investigation for potential therapies for patients with POAG, which could be considered in future glaucoma research.

## 5. Disclosure

The funding organizations had no role in the design or conduct of this research.

## Figures and Tables

**Table 1:** Primer pairs are used for a reverse transcription-polymerase chain reaction.

Gene	Sense primer	Antisense primer	Probe (bp)
*β*-actin	5′-CCTGGCACCCAGCACAAT-3′	3′-GGGCCGGACTCGTCATAC-5′	144
LC3-II	5′-GCCTTCTTCCTGTTGGTGAA-3′	3′-CTGGGAGGCATAGACCATGT-5′	117
Beclin1	5′-TGTCACCATCCAGGAACTCA-3′	3′-CCTGGCGAGGAGTTTCAATA-5′	119
MMP-2	5′-ATGACAGCTGCACCACTGAG-3′	3′-AGTTCCCACCAACAGTGGAC-5′	126

## Data Availability

All data relevant to this study are available upon reasonable request from the corresponding author.

## Supplementary Materials

Supplementary Materials SFigure 1: identification of trabecular meshwork cells (200×). A: fibronectin (FN) staining. B: laminin (LM) staining. C: neuron-specific enolase (NSE) staining. D: vimentin staining. E: factor VIII-associated antigen staining. F: negative control staining. SFigure 2: the effect of 3-MA and starvation on cell proliferation was analyzed by the MTT assay. The cell inhibition rate from three independent experiments is expressed as the mean ± standard deviation (SD), *n* = 3. A: the growth curve of trabecular meshwork cells for 3-MA treatment at 2.5, 5, 10, and 20 mM. B: the growth curve of trabecular meshwork cells for starvation treatment at 6, 12, 18, 24, and 36 h. SFigure 3: effects of 3-MA and starvation on the autophagy of trabecular meshwork cells were evaluated roughly by monodansylcadaverine (MDC)-labeled cells in MDC fluorescent staining. A: group control. B: group 3-MA (5 mM). C: group starvation.
