# Supplementary material for: Autophagy Mediates MMP-2 Expression in Glaucomatous Trabecular Meshwork Cells
Source: J Ophthalmol. 2022 Sep 10;2022:6026464. doi: 10.1155/2022/6026464 (PMC9536984; doi:10.1155/2022/6026464)
Supplement: Supplementary Materials — SFigure 1: identification of trabecular meshwork cells (200×). A: fibronectin (FN) staining. B: laminin (LM) staining. C: neuron-specific enolase (NSE) staining. D: vimentin staining. E: factor VIII-associated antigen staining. F: negative control staining. SFigure 2: the effect of 3-MA and starvation on cell proliferation was analyzed by the MTT assay. The cell inhibition rate from three independent experiments is expressed as the mean ± standard deviation (SD), n = 3. A: the growth curve of trabecular meshwork cells for 3-MA treatment at 2.5, 5, 10, and 20 mM. B: the growth curve of trabecular meshwork cells for starvation treatment at 6, 12, 18, 24, and 36 h. SFigure 3: effects of 3-MA and starvation on the autophagy of trabecular meshwork cells were evaluated roughly by monodansylcadaverine (MDC)-labeled cells in MDC fluorescent staining. A: group control. B: group 3-MA (5 mM). C: group starvation. [file 6026464.f1.docx]

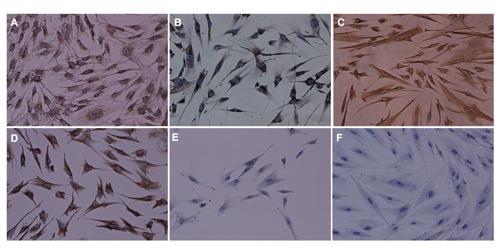


SFigure 1. Identification of trabecular meshwork cells (200×). **A**: Fibronectin (FN) staining. **B**: Laminin (LM) staining. **C**: Neuron-specific enolase (NSE) staining. **D**: Vimentin staining. **E**: Factor VIII-associated antigen staining. **F**: Negative control staining.


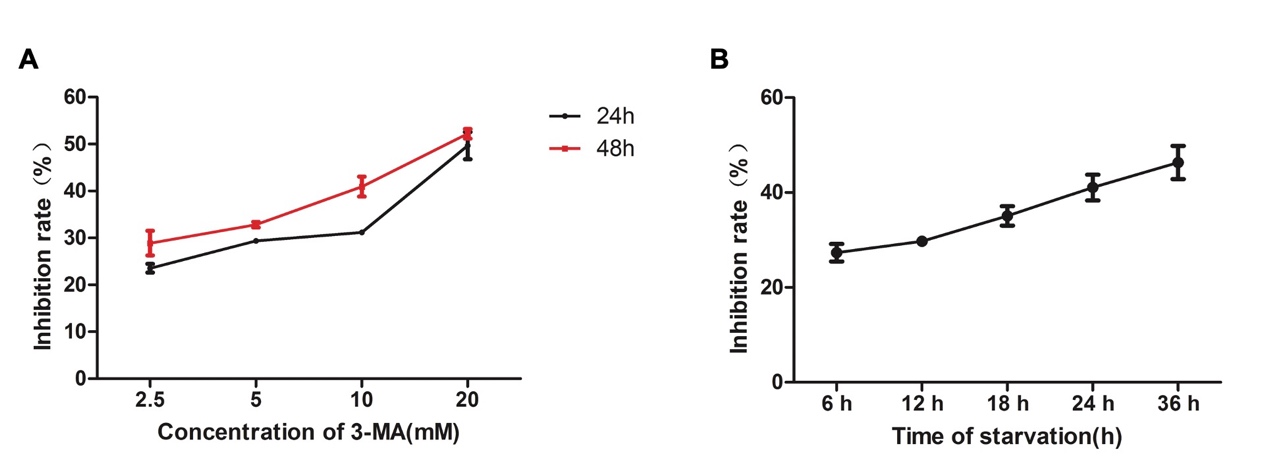
SFigure 2. The effect of 3-MA and starvation on cell proliferation analyzed by MTT assay. The cells inhibition rate from three independent experiments is expressed as mean ± standard deviation (SD), n = 3. **A**: Growth curve of trabecular meshwork cells for 3-MA treatment at 2.5, 5, 10, 20mM. **B**: Growth curve of trabecular meshwork cells for starvation treatment at 6, 12, 18, 24 and 36 h.


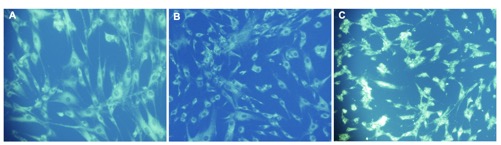
SFigure 3. Effects of 3-MA and starvation on the autophagy of trabecular meshwork cells were evaluated roughly by Monodansylcadaverine (MDC) labeled cells in MDC fluorescent staining. **A**: Group control. **B**: Group 3-MA (5 mM). **C**: Group starvation.
